# Self-repair in a bidirectionally coupled astrocyte-neuron (AN) system based on retrograde signaling

**DOI:** 10.3389/fncom.2012.00076

**Published:** 2012-09-26

**Authors:** John Wade, Liam McDaid, Jim Harkin, Vincenzo Crunelli, Scott Kelso

**Affiliations:** ^1^Intelligent Systems Research Center, School of Computing and Intelligent Systems, University of UlsterDerry, Northern Ireland, UK; ^2^Neuroscience Division, Cardiff School of Biosciences, University of CardiffCardiff, UK; ^3^Center for Complex Systems and Brain Sciences, Florida Atlantic UniversityBoca Raton, FL, USA

**Keywords:** astrocyte, calcium wave, endocannabinoid, IP3, neuron, self-repair, synapse

## Abstract

In this paper we demonstrate that retrograde signaling via astrocytes may underpin self-repair in the brain. Faults manifest themselves in silent or near silent neurons caused by low transmission probability (PR) synapses; the enhancement of the transmission PR of a healthy neighboring synapse by retrograde signaling can enhance the transmission PR of the “faulty” synapse (repair). Our model of self-repair is based on recent research showing that retrograde signaling via astrocytes can increase the PR of neurotransmitter release at damaged or low transmission PR synapses. The model demonstrates that astrocytes are capable of bidirectional communication with neurons which leads to modulation of synaptic activity, and that indirect signaling through retrograde messengers such as endocannabinoids leads to modulation of synaptic transmission PR. Although our model operates at the level of cells, it provides a new research direction on brain-like self-repair which can be extended to networks of astrocytes and neurons. It also provides a biologically inspired basis for developing highly adaptive, distributed computing systems that can, at fine levels of granularity, fault detect, diagnose and self-repair autonomously, without the traditional constraint of a central fault detect/repair unit.

## Introduction

Traditionally, communication, information transfer, and plasticity within the brain have been the sole province of the chemical synapses made by pre- and post-synaptic neurons. However, current research has challenged this view of synaptic physiology where it is now believed that astrocytes, a type of glial cell found in the central and peripheral nervous system can encapsulate ~10^5^ synapses and can connect to multiple neighboring neurons (Bushong et al., 2002; Halassa et al., 2007); this intimate connection between astrocytes and neurons is named the *tripartite synapse* (Araque et al., 1999).

Although astrocytes cannot elicit propagating action potentials (APs) like neurons do, their “unit of excitation” is the transient increase in intracellular calcium (Ca^2+^) levels that is elicited by various neurotransmitters (e.g., glutamate, ATP, GABA, etc.) following binding to their respective receptors on the astrocytic membrane. These astrocytic Ca^2+^ transients in turn lead to astrocytic release of transmitters (often referred to as “gliotransmitters”) and to propagating Ca^2+^ waves (Dani et al., 1992; Porter and McCarthy, 1996). Although the propagation of intracellular Ca^2+^ is not fully understood, the process is believed to be facilitated by signaling proteins between microdomain clusters of inosotil 1, 4, 5-trisphosphate Receptors (IP_3_Rs) (Weerth et al., 2007; Agulhon et al., 2008). Astrocytes also communicate in a feedback mode with neurons. In response to elevated levels of intracellular Ca^2+^, astrocytes can release gliotransmitters such as glutamate which bind to extrasynaptic receptors on the post synaptic neuron (Corlew et al., 2008). This bidirectional communication between astrocytes and neurons results in various forms of synaptic modulation.

Alongside the astrocyte's role in synaptic regulation they are also implicated in synaptogenesis and synaptic maintenance (Slezak and Pfrieger, 2003) which may have a role in how the brain carries out repairs. This is further supported by the recent finding that astrocytes possess binding sites for endocannabinoids, a type of retrograde messenger released post-synaptically during neuronal depolarization (Alger, 2002). Similar to neurotransmitter uptake, this leads to the oscillation of Ca^2+^ levels within the astrocyte and the release of glutamate. Such a signaling pathway acts to modulate the transmission probability (PR) of the synapse and is a potential candidate for self-repair of damaged or low PR synapses (Navarrete and Araque, 2010).

Understanding the mechanisms that underpin the brain's distributed and fine-grained repair capability still however remains a key challenge. To this end, we propose a simple computational model for self-repair based on bidirectional interactions between astrocytes and neurons (Araque et al., 2001; Perea and Araque, 2005). This paper is an extension of our previous work (Wade et al., 2011a,b) but now demonstrates self-repair through modulation of synaptic release PR where we consider a fault to be a condition which results in silent or near silent neurons caused by low transmission PR synapses; the enhancement of the transmission PR of a “faulty” synapse by retrograde signaling can enhance the transmission PR (repair) and we show that a key mechanism underlying PR is indirect signaling through retrograde messengers such as endocannabinoids.

## Materials and methods

### Endocannabinoid mediated self-repair

Upon the arrival of an AP at the pre-synaptic axon, neurotransmitter (glutamate) is released across the cleft and binds to receptors on the post-synaptic dendrite causing a depolarization of the post-synaptic neuron. When the post-synaptic neuron is sufficiently depolarized (e.g., emits an output spike), voltage gated channels on the dendrite allow the influx of Ca^2+^ into the dendrite causing endocannabinoids to be synthesized and subsequently released from the dendrite. However, the exact release machinery underlying this process is not fully understood (Alger, 2002). Endocannabinoids are a type of retrograde messenger which travel back from the post-synaptic terminal to the pre-synaptic terminal. The release of 2-arachidonyl glycerol (2-AG), a type of endocannabinoid, is known to feed back to the pre-synaptic terminal in two ways:

1. **Directly:** 2-AG binds directly to type 1 Cannabinoid Receptors (CB1Rs) on the pre-synaptic terminal. This results in a decrease in transmission PR and is termed Depolarization-induced Suppression of Excitation (DSE) (Alger, 2002).
2. **Indirectly:** 2-AG binds to CB1Rs on an astrocyte which enwraps the synapse increasing IP_3_ levels within the astrocyte and triggering the intracellular release of Ca^2+^. This results in the astrocytic release of glutamate which binds to pre-synaptic group I metabotropic Glutamate Receptors (mGluRs). Such signaling results in an increase of synaptic transmission PR termed e-SP (Navarrete and Araque, 2010).

Experimental evidence shows that local synapses (i.e., synapses where post-synaptic firing results in both direct and indirect signaling) exhibit DSE and PR is reduced by ~50%. This is thought to be a result of the direct signaling pathway overpowering the indirect pathway. The direct signaling pathway is very local since 2-AG can only travel ~20 nm within the extracellular fluid and therefore binds only with a few neighboring synapses. The indirect signaling pathway is however far reaching and can affect distant synapses (Navarrete and Araque, 2010). Since astrocytes can enwrap very many (~10^5^) synapses and contact ~6 neurons within the cortex and hippocampus (Halassa et al., 2007), the indirect signaling pathway has the potential to reach many synapses via the astrocyte. Distal silent synapses expressing indirect signaling via the astrocyte only, exhibit e-SP where PR increases by ~200% (Navarrete and Araque, 2010). The repair mechanism proposed and modeled utilizes both DSE and the e-SP signal via an astrocyte to increase PR in neighboring synapses.

Given the known properties of endocannabinoids for the modulation of synaptic transmission PR, we hypothesize here that the indirect signaling pathway is the catalyst for self-repair of damaged or low PR synapses. For instance, consider the case where a synapse is damaged with a low PR insufficient to cause post-synaptic activity. Because this neuron is not emitting 2-AG its associated synapses will experience an increase in PR due to the release of 2-AG from neighboring neurons. This messenger causes the release of glutamate from the astrocyte cell activating type I mGluRs in the pre-synaptic terminal.

The proposed self-repairing model builds on two biophysically motivated models which describe the interactions between astrocytes and neurons in a tripartite synapse: namely the gatekeeper model (Volman et al., 2007) and the Nadkarni and Jung model (Nadkarni and Jung, 2004, 2007). Both of these models use the Li-Rinzel model (Li and Rinzel, 1994) to describe the evolution of synapse driven Ca^2+^ within the astrocyte; Ca^2+^ regulates synapse transmission via the release of glutamate which binds to pre-synaptic receptors.

### Endocannabinoid dynamics

**Figure 1:**
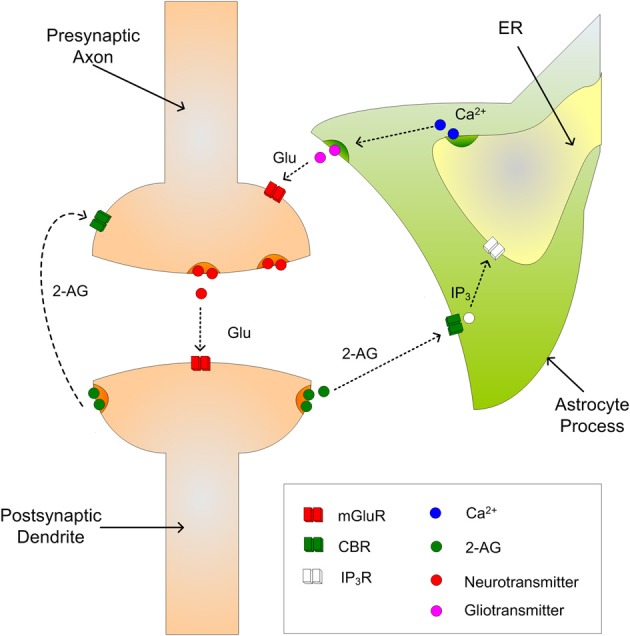
**The tripartite synapse showing indirect and direct signaling of 2-AG**.

The gatekeeper (Volman et al., 2007) and Nadkarni and Jung (2004, 2007) models describe the interaction of astrocytes and neurons via the tripartite synapse. In a tripartite synapse an astrocyte process connects with the axon and dendrite of the pre- and post-synaptic neurons and is sensitive to the neurotransmitters within the extracellular fluid in the synaptic cleft (Araque et al., 1999). However, the tack taken in the current work is to model the astrocytes sensitivity to 2-AG instead of neurotransmitter. Figure 1 illustrates a tripartite synapse with 2-AG signaling.

When neurotransmitter, e.g., glutamate, is released into the synaptic cleft and the post-synaptic neuron is sufficiently depolarized, 2-AG is released from the dendrite and binds to CB1Rs on the astrocyte process. This in turn initiates the creation and release of IP_3_ into the cytoplasm of the astrocyte which subsequently binds to IP_3_Rs on the Endoplasmic Reticulum (ER); the ER is a long network of tubes and vesicles used to store calcium within the cell (Kurosinski and Gotz, 2002). The binding of IP_3_ with IP_3_Rs opens channels that allow the release of Ca^2+^ from the ER in to the cytoplasm. While individual Ca^2+^ releases are incapable of propagating intracellularly, several releases can raise Ca^2+^ levels in the cytoplasm beyond a threshold and an oscillating Calcium Induced Calcium Release (CICR) propagation can be observed (Marchant et al., 1999); the threshold is believed to be of the order 0.2–0.4 μM (Bezprozvanny et al., 1991). The increase in cytosolic Ca^2+^ then causes the release of gliotransmitter back into the synaptic cleft which binds to pre-synaptic group I mGluRs, i.e., indirect signaling. The 2-AG also binds directly (direct signaling) to the pre-synaptic CB1Rs which causes a decrease in PR.

To model 2-AG release we assume each time a post synaptic neuron fires, 2-AG is released and can be described as follows:

(1) $$\frac{d(\text{AG})}{dt}=\frac{-\text{AG}}{\tau_{\text{AG}}}+r_{\text{AG}}\delta (t-t_{\text{sp}})$$

where AG is the quantity of 2-AG, τ_AG_ is the decay rate of 2-AG, *r*_AG_ is the 2-AG production rate (= 0.8 μMs^−1^) and *t*_sp_ is the time of the post-synaptic spike. To our best knowledge no data are available in the literature on the magnitude of τ_AG_ and therefore we have assumed that the lifetime of 2-AG is consistent with other time constants: for example the effects of e-SP are known to have a rise time of ~100 s and a decay time of ~200 s (Navarrete and Araque, 2010), therefore a value of 10 s is assumed for τ_AG_.

### Calcium dynamics

In the present model, 2-AG binds to CB1Rs on the astrocyte process and the generation of IP_3_ is achieved in a similar manner to the gatekeeper model (Volman et al., 2007). This process is assumed to be dependent on the amount of 2-AG released. The generation of IP_3_ is given by:

(2) $$\frac{d({\text{IP}}_{3})}{dt}=\frac{{\text{IP}}_{3}^{*}-{\text{IP}}_{3}}{\tau_{\text{ip}3}}+r_{\text{ip}3}\text{AG}$$

where IP_3_ is the amount within the cytoplasm, *r*_ip3_ is the production rate of IP_3_ and is set at 0.5 μMs^−1^, IP^*^_3_ is the baseline of IP_3_ within the cytoplasm when the cell is in a steady state and receiving no input, and τ_ip3_ is the IP_3_decay rate.

The Li-Rinzel model uses three channels to describe the Ca^2+^ dynamics within a cell: *J*_pump_ models how Ca^2+^ is stored within the ER by pumping Ca^2+^ out of the cytoplasm into the ER via Sarco-Endoplasmic-Reticulum Ca^2+^-ATPase (SERCA) pumps, *J*_leak_ describes Ca^2+^ leakage into the cytoplasm from the ER and *J*_chan_ models the opening of Ca^2+^ channels by the mutual gating of Ca^2+^ and IP_3_ concentrations. Since the model only considers the case of a single cell which exists in a Ca^2+^-free extracellular environment, no account is taken of any Ca^2+^ flux across the cell membrane (De Pittà et al., 2008). The Li-Rinzel model is described using the following equations (a full derivation of these equations is provided by De Pittà et al., 2009):

(3) $$\frac{d({\text{Ca}}^{2+})}{dt}=J_{\text{chan}}\left({\text{Ca}}^{2+},h,{\text{IP}}_{3}\right)+J_{\text{leak}}\left({\text{Ca}}^{2+}\right)-J_{\text{pump}}\left({\text{Ca}}^{2+}\right)$$

(4) $$\frac{dh}{dt}=\frac{h_{\infty }-h}{\tau_{h}}$$

where *J*_chan_ is the IP_3_ and Ca^2+^-dependent Ca^2+^ release, *J*_pump_ is the amount of Ca^2+^ pumped from the cytoplasm into the ER via the SERCA pumps, *J*_leak_ is the Ca^2+^ which leaks out of the ER and *h* is considered to be the fraction of activated IP_3_Rs. The parameters *h*_∞_ and τ_*h*_ are given by:

(5) $$h_{\infty }=\frac{Q_{2}}{Q_{2}+{\text{Ca}}^{2+}}$$

and

(6) $$\tau_{h}=\frac{1}{a_{2}\left(Q_{2}+{\text{Ca}}^{2+}\right)}$$

where

(7) $$Q_{2}=d_{2}\frac{{\text{IP}}_{3}+d_{1}}{{\text{IP}}_{3}+d_{3}}$$

The description of the *J*_chan_ channel is given by:

(8) $$J_{\text{chan}}=r_{\text{C}}m_{\infty }^{3}n_{\infty }^{3}h^{3}\left(C_{0}-\left(1+c_{1}\right){\text{Ca}}^{2+}\right)$$

where *r*_*C*_ is the maximal CICR rate, *C*_0_ is the total free Ca^2+^ cytosolic concentration, *C*_1_ is the ER/cytoplasm volume ratio and *m*_8_ and *n*_8_ are the IP_3_ Induced Calcium Release (IICR) and CICR channels, respectively, and are given by:

(9) $$m_{\infty }=\frac{{\text{IP}}_{3}}{{\text{IP}}_{3}+d_{1}}$$

and

(10) $$n_{\infty }=\frac{{\text{Ca}}^{2+}}{{\text{Ca}}^{2+}+d_{5}}$$

The remaining channels are given by:

(11) $$J_{\text{leak}}=r_{\text{L}}\left(C_{0}-\left(1+c_{1}\right){\text{Ca}}^{2+}\right)$$

and

(12) $$J_{\text{pump}}=v_{\text{ER}}\frac{{\left({\text{Ca}}^{2+}\right)}^{2}}{k_{\text{ER}}^{2}+{\left({\text{Ca}}^{2+}\right)}^{2}}$$

where *r*_L_ is the Ca^2+^ leakage rate, *v*_ER_ is the maximum SERCA pump uptake rate and *k*_ER_ is the SERCA pump activation constant.

### Endocannabinoid-mediated synaptic depression/potentiation (DSE/e-SP)

There is no clear consensus in the literature on the relationship between DSE and the level of 2-AG released by the post synaptic neuron. In the present case we assume a linear correspondence given by:

(13) $$\text{DSE}=\text{AG}\times K_{\text{AG}}$$

where AG is the amount of 2-AG released by the post-synaptic neuron and is found from Equation (1) and *K*_AG_ (= −4000) is a scaling factor used to convert the level of 2-AG into the desired negative range. The intracellular astrocytic calcium dynamics are used to regulate the release of glutamate from the astrocyte which drives e-SP. To model this release, we assume when Ca^2+^ crosses the CICR threshold from below that a quantity of glutamate targeting group I mGluRs is released every 300 ms and is given by:

(14) $$\frac{d(\text{Glu})}{dt}=\frac{-\text{Glu}}{\tau_{\text{Glu}}}+r_{\text{Glu}}\delta (t-t_{\text{Ca}})$$

where Glu is the quantity of glutamate, τ_Glu_ is the decay rate of glutamate (= 100 ms), *r*_*Glu*_ is the glutamate production rate (= 10 μMs^−1^) and *t*_Ca_ is the time of the Ca^2+^ threshold crossing. It is believed that Ca^2+^ oscillations can be initiated within discrete microdomains (Panatier et al., 2011) and can be localized or propagated intracellularly by activating neighboring microdomains of storage (Pasti et al., 1997; Carmignoto, 2000; Weerth et al., 2007; Agulhon et al., 2008; Di Castro et al., 2011). Therefore, the level of Ca^2+^ within the cell differs depending on spatial location. However, for simplicity in the present model we assume that the instantaneous level of Ca^2+^ remains the same everywhere; therefore the release of glutamate is also assumed to be instantaneous.

To model e-SP we use the following:

(15) $$\tau_{\text{eSP}}\frac{d(\text{eSP})}{dt}=-\text{eSP}+m_{\text{eSP}}\text{Glu}(t)$$

where τ_eSP_ is the decay rate of e-SP (= 40 s) and *m*_eSP_ is a weighting constant (= 55 × 10^3^) used to control the height of e-SP. From Equation (15) it is clear that the level of e-SP is dependent on the quantity of glutamate released by the astrocyte.

Parameters and initial variable values used throughout this paper can be found in Tables A1–A3. The initial variable values represent the system in a quiescent state. Note that initial values for *h* and Ca^2+^ were found experimentally by initializing Ca^2+^ and *h* to 0.5 and 0.06 μM, respectively. The model was then simulated with IP_3_ levels clamped at 0.16 μM (IP^*^_3_) until Ca^2+^ and *h* stabilized.

### Self repairing paradigm

**Figure 2:**
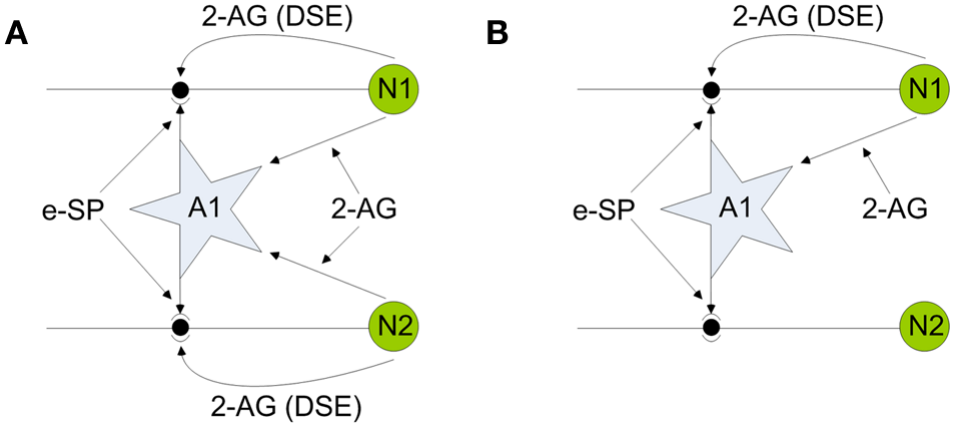
**Network fragments illustrating endocannabinoid mediated self-repair. (A)** Network before fault. **(B)** Network after fault. Note 2-AG is a local signal associated with each synapse connected to either neuron N1 or N2, whereas e-SP is a global signal associated with all synapses connected to the astrocyte A1.

Our self repairing paradigm is proposed with reference to Figures 2(A) and (B), where **(A)** shows a simple SNN network fragment with no faults and **(B)** show the same network where the PR has been reduced (to simulate a fault) in the synapse associated with the post-synaptic neuron N2.

To illustrate the self repairing concept we first consider the case where both synapses are healthy, as depicted in Figure 2A: both direct and indirect feedback signals compete at each synapse to alter PR and enable a stable state to be reached (signal conditions for the non-faulty state will be verified later on). In Figure 2B a fault is introduced into the synapse associated with N2 and hence both direct and indirect retrograde feedback from neuron N2 ceases. This creates an imbalance in PR at the synapse associated with N2 with the result that PR increases to restore functionality due to indirect retrograde feedback from N1. This is the self-repairing mechanism proposed here and is verified by the computational results shown later in Figure 5. Our simulations demonstrate that when faults occur in synapses associated with a neuron, indirect feedback from other neurons implements repair by increasing the PR across all synapses (faulty and non-faulty) associated with the neuron to restore the original functionality.

### Neuron model

Although many neuron models exist such as the Hodgkin–Huxley model (Hodgkin and Huxley, 1952), the simplified counterparts such as (FitzHugh, 1961; Nagumo et al., 1962; Morris and Lecar, 1981) are often preferred (Gerstner and Naud, 2009). Nevertheless, these models are still computationally expensive and require a great deal of parameter tuning. However, one of the most widely used neural models is the Leaky Integrate and Fire (LIF) model (Gerstner and Kistler, 2002) which has relatively few parameters (Gerstner and Naud, 2009) and requires relatively little computational effort due to its simplistic nature. Consequently the LIF is more suited to large network simulations (Bugmann, 1997). The LIF model used in this work is the passive model (Gerstner and Kistler, 2002) described by:

(16) $$\tau_{m}\frac{dv}{dt}=-v(t)+R_{m}\sum_{i=1}^{n}I_{\text{syn}}^{i}\left(t\right)$$

where τ_*m*_ is the membrane time constant, *v* is the membrane potential and *R*_*m*_ is the membrane resistance and *I*^*i*^_syn_ is the current injected to the neural membrane at synapse *i*. If *v* is greater than the firing threshold (*v*_th_) then *v* is clamped at 0V for 2 ms thereby implementing the refractory period of the neuron.

### Synapse model

The synapse model used here is probabilistic-based where each time a pre-synaptic spike is presented to the synapse a uniformly distributed pseudorandom number generates a number between 0 and 1 (*rand*). If the value of the random number is less than or equal to the release PR a fixed current *I*_inj_ (= 6650 pA) is injected into the LIF given by:

(17) $$I_{\text{syn}}^{i}(t)=\{\begin{array}{c} I_{\text{inj}}\text{ rand}\leq \text{PR}\\ 0\text{ rand}>\text{PR} \end{array}$$

All synapse and neuron parameters can be found in Table A3. If we now consider the case where the network is functioning without fault (Figure 2A) then the associated PR of each associated synapse is governed by the following:

(18) $$\text{PR}(t)=\left(\frac{\text{PR}\left(t_{0}\right)}{100}\times \text{DSE}(t)\right)+\left(\frac{\text{PR}\left(t_{0}\right)}{100}\times \text{eSP}(t)\right)$$

where PR(*t*_0_) is the initial PR of each associated synapse. The variables of DSE and e-SP have been tuned so that Equation (18) results in an overall depression of each of the synapses by ~50% in accordance with Navarrete and Araque (2010). However, if we consider the case in which a number of synapses become faulty and therefore DSE decreases, then the depression of each synapse is decreased as e-SP starts to overpower DSE. In extreme faults the PR is given by Equation (19), which is ~200% and the associated neuron (N2 in Figure 2B) exhibits a significantly reduced firing rate with no appreciable direct or indirect impact on the synapse.

(19) $$\text{PR}\to \left(\frac{\text{PR}\left(t_{0}\right)}{100}\times \text{eSP}\left(t\right)\right)$$

However, indirect feedback from N1 via the astrocyte increases e-SP, and *PR* at the synapses is proportionally increased. We view this as a repair mechanism. Therefore, the value of PR at time *t*, PR(*t*), is a percentage of the initial value of PR(*t*_0_) and is governed by the indirect signaling pathway between the astrocyte and neuron (e-SP). Note that when a fault is simulated the value PR(*t*_0_) is set to the fault PR value of the synapse.

## Results

Here we present results of simulations that highlight the dynamics of our model and demonstrate how self-repair can occur at synapses. In these simulations the network shown in Figure 2 is used with each neuron receiving input from 10 synapses. All synapses have an initial PR of 0.5 and are simulated with a unique Poisson distributed spike train with an average firing rate of 10 Hz. The Matlab 2009a simulation environment was employed throughout and the Euler method of integration with a fixed time step of Δ*t* = 1 ms was used in all simulations. Results remained unchanged using a time step of Δ *t* = 0.1 ms (data not shown).

### Simulation with no fault

**Figure 3:**
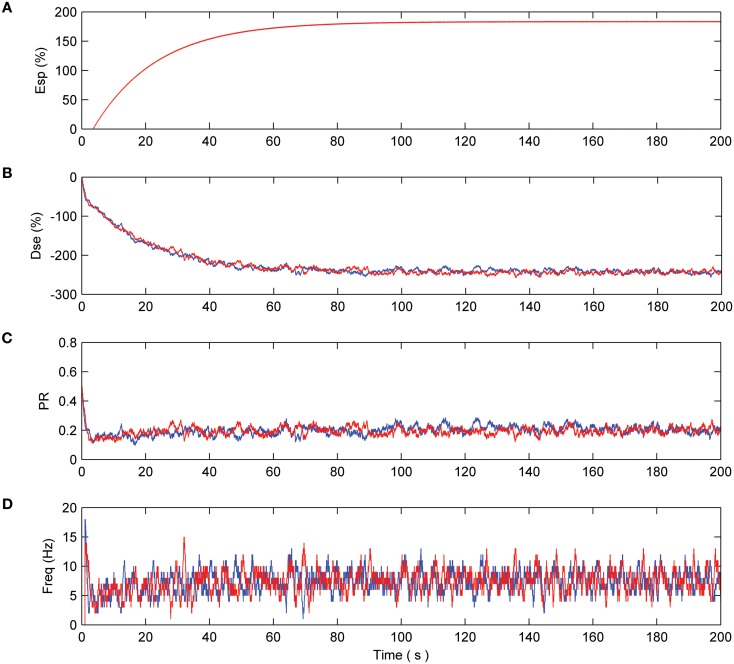
**Network with no fault. (A)** e-SP function of both N1(blue) and N2 (red). Since e-SP is a global function and relates to all synapses connected by the astrocyte it is the same for N1 and N2 **(B)** DSE function of N1 and N2. Since DSE is only local to all synapses connected to a neuron, DSE is different in N1 and N2 and is driven by the output of each neuron. **(C)** The probability of a synapse connected to N1 and N2. This probability is the summation of e-SP and DSE presented to the neuron. Note how the probability is reduced by ~50% which results in an overall reduction of the firing rate of N1 and N2 as seen in **(D)**.

Consider the case where neurons N1 and N2 are firing as a result of the presented pre-synaptic stimuli and coupling with the astrocyte occurs via the 2-AG signal. This causes the release of glutamate which acts on mGluRs receptors on the pre-synaptic terminals of both neurons (see Figure 2). Both neurons are simulated for a period of 200 s. From Figure 3(A) it can be seen that the e-SP function is global to both N2 and N2 while DSE is local to the synapses associated with the individual neurons. Thus, the synapse of N1 receives a different DSE signal to that of N2 [Figure 3(B)]. Figure 3(C) presents the PR of a synapse associated with N1 (N2). Note how PR is reduced by ~50% due to the summation of e-SP and DSE at the synapse. Furthermore each of the synapses of N1 and N2 receives the same PR value as depicted in **(C)**. Finally, Figure 3(D) depicts the average firing rates of both neurons, where it can be seen that the firing rates of both neurons are reduced from the initial firing rate. This results from the overall depression of all synapses by ~50%.

### Simulation partial fault (moderate reduction of PR)

**Figure 4:**
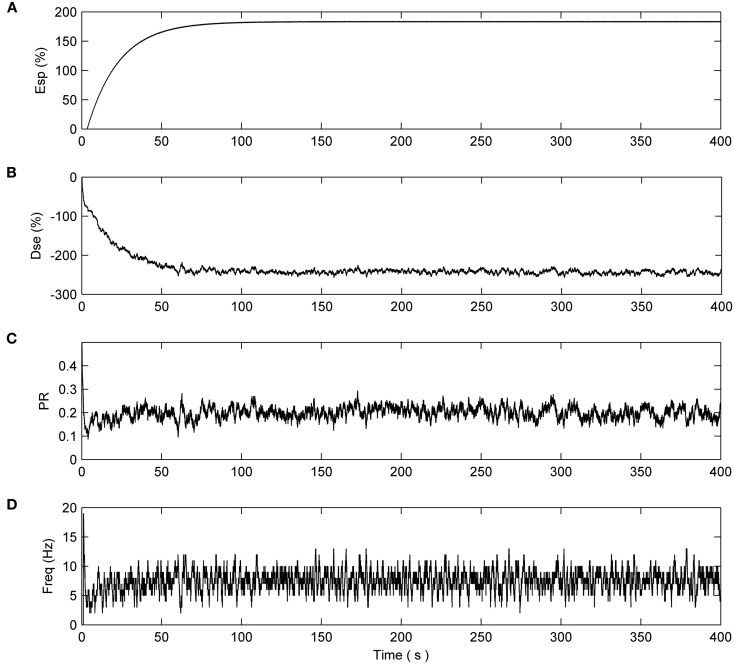
**Network with partial fault (N1).** No synapse connected to N1 have a fault. **(A)** e-SP signal. **(B)** DSE signal. **(C)** PR of the first synapse connected to N1. The remaining synapses connected to N1 have the same PR dynamics. **(D)** Average firing rate of N1.

Now consider the case where N1 and N2 are stimulated by multiple synapses (10 in this example) and that the PR associated with 80% of the synapse of N2 is deliberately reduced (simulating a fault) to 0.1 after 200 s. From Figure 4 we can see that N1, as expected, is unaffected and the PR values of all synapses connected to N1 are depressed at ~50% of their initial value.

**Figure 5:**
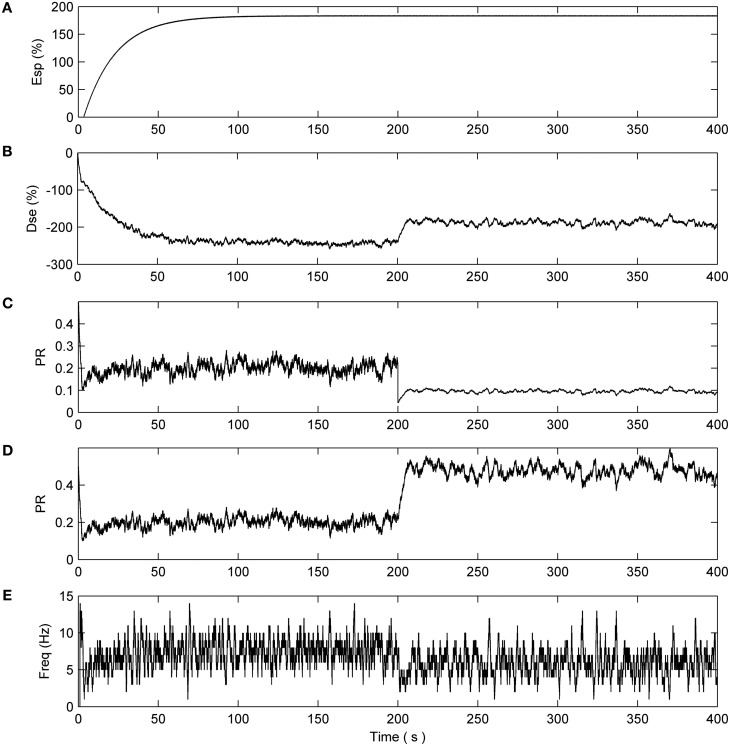
**Network with 80% of N2 synapses with PR reduced to 0.1 after 200 s. (A)** e-SP signal. **(B)** DSE signal. **(C)** PR of the first synapse connected to N2. **(D)** PR of the non faulty synapses. **(E)** Average firing rate of N2 showing the rate falling off at 200 s and increasing thereafter due to the hypothesized repair process.

This is not the case with synapses associated with N2 (Figure 5). After 200 s the reduction in PR of the faulty synapses causes a decrease in the output firing rate of N2 thereby reducing the associated DSE signal. This creates an imbalance in the dynamics and e-SP is allowed to enhance all synapses connected to N2. Note how the PR of the remaining healthy synapses (Figure 5D) is enhanced to a greater extent when compared to the faulty synapses (Figure 5C) suggesting that the introduction of faults perturbs the balance between DSE and e-SP such that an overall increase in PR results primarily in the healthy synapses. This is akin to redistributing the PR across the remaining 20% of healthy synapses. Indicative of the proposed repair process, Figure 5D shows that the average firing rate of N2 falls after 200 s but then increases again after a period of time due to the redistribution of PR across all synapses (repair process).

### Simulation with complete fault (PR reduced to 0)

**Figure 6:**
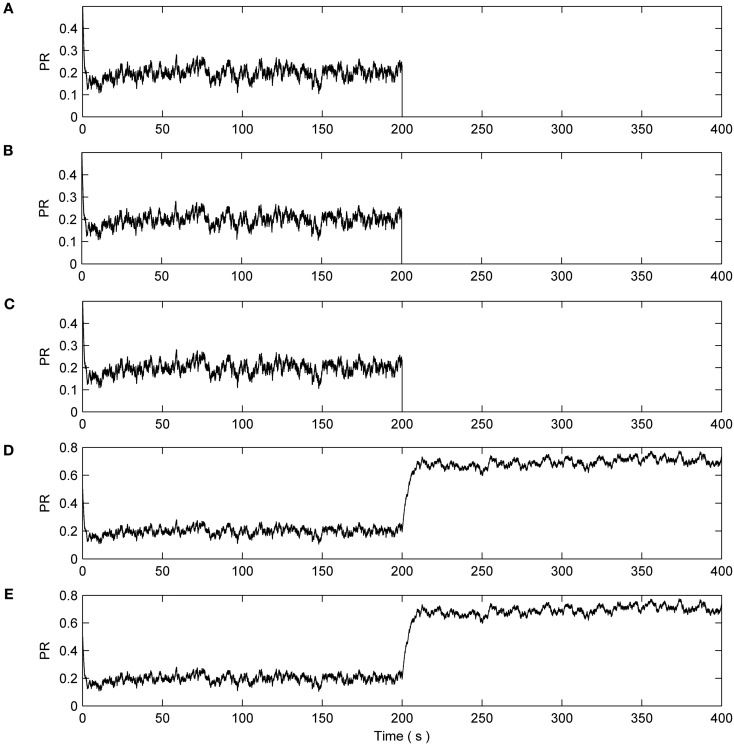
**PR values of synapses of N2. (A–C)** Show three faulty synapses where the fault is induced at time 200 s. **(D,E)** Demonstrates the PR of the remaining non faulty synapses increasing to compensate for the net loss of **(A–C)**, thereby restoring the functionality of N2.

Next we consider the case when 8 out of the 10 synapses connected to N2 exhibit a catastrophic failure such as their connecting axons being severed due to injury. This is simulated by reducing the associated PRs to 0 at 200 s. The total length of this simulation is 400 s. Again N1 is unaffected and the synapses have a reduction of ~50% as in the previous cases (data not shown). Figure 6 presents the PR from 50% of the synapses connected to N2: eight synapses had induced faults while two were left to function normally. It can be seen from plots A–C of Figure 6 that no repair occurs, i.e., PR = 0. However, the PR values of the remaining two non-faulty synapses were again significantly increased beyond their initial value of 0.5 as repair takes place. Once again, the faults are tolerated by an increase in PR at the non-faulty synapses.

**Figure 7:**
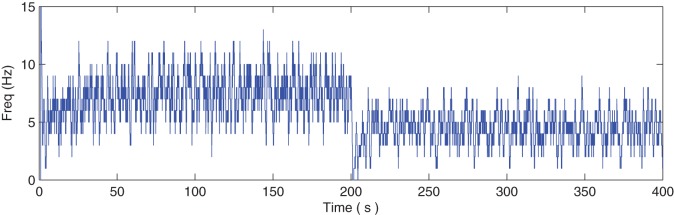
**Average firing rate of N2.** Note how the output frequency of N2 falls to zero at 200 s as a result of the catastrophic failure of 80% of the synapses connected to N2. As the self-repair mechanism kicks in and increases the PR value of non-faulty synapses, the output frequency of N2 also increases.

Figure 7 describes the average firing rate of N2 where it can be seen that the firing rate falls to ~0 Hz at 200 s but recovers after a few seconds (albeit to a lower firing rate) when repair has taken place. Again, the repair shows partial recovery of the firing rate.

### Simulation with no e-SP

The results of the previous experiments suggest that when synapses within the Astrocyte-Neural Network become faulty, repairs are implemented by enhancement of PR at other non-faulty synapses. To prove that no other mechanism is responsible for repair we repeat the above experiment in the absence of the astrocyte cell, i.e., without an indirect feedback signal (e-SP). The results showed a decrease in PR of all synapses of N1 and N2 in the order of ~200% due to direct feedback. At the onset of induced faults at 200 s, the functioning synapse of N2 exhibited an increase in PR due to the reduction in DSE associated with N2's drop in firing activity (data not shown). Such PR increase is much less than that found in the previous simulation and is ineffective as a repair mechanism.

### Calcium dynamics

**Figure 8:**
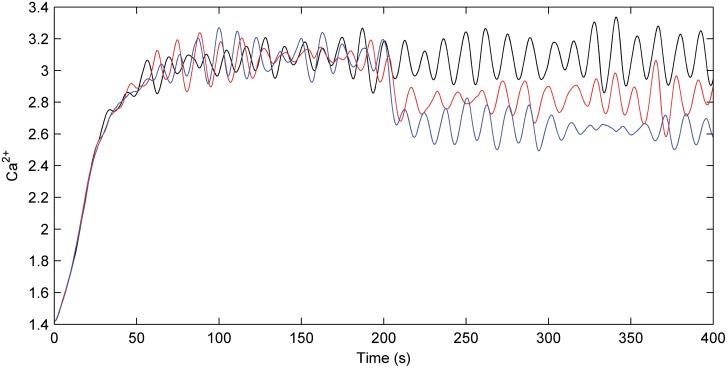
**Astrocyte calcium dynamics.** Calcium dynamics for no fault (black), partial fault (red), and catastrophic fault (blue). When a fault occurs the Ca^2+^ levels drop within the astrocyte. However, as a result of the e-SP produced by the astrocyte, Ca^2+^ levels are not reduced substantially and oscillations continue.

Figure 8 describes the astrocyte calcium dynamics for *no fault*, *partial fault*, and *catastrophic conditions* as described by the previous three simulations. Note there is a reasonable constant Ca^2+^ oscillation when there is no fault; however, when a fault is induced at 200 s the overall calcium levels drop and the oscillations continue. As long as the overall Ca^2+^ level remains above the threshold the astrocyte will release glutamate in an attempt to reinforce the PR of all synapses.

## Discussion

This work was motivated by the need to understand how the brain regulates itself to cope with injury. Exploiting the biological adaptive/repair mechanisms of the brain (Stevens, 2008) would provide a novel approach to fault tolerant computing, which goes beyond existing capabilities where reliable computations could then be realized using neural networks (Patterson et al., 2012), instead of traditional von Neumann computing architectures. Neural networks offer a fine-grained distributed computing architecture that captures to some degree high levels of parallel processing in the brain. The fine-grained parallelism provides the framework that enables fault tolerance to be realized at very low levels of granularity; i.e., computations are mapped across many neuron clusters permitting a “scattering” of faults without a significant level of computing degradation. However, high levels of parallelism are not the only contributor to fault tolerance as the brain uses key repair mechanisms to continually adapt to conditions via re-wiring pathways to cope with decaying or damaged neurons (Slezak and Pfrieger, 2003). Therefore, we believe our model is the first step in addressing the key challenge which is to understand the mechanisms that underpin the brain's distributed and fine-grained repair capability. From a purely engineering point of view, modeling the interaction between cells at a network level may lead to a truly brain-inspired paradigm for fault tolerant computing beyond current self-repairing hardware architectures (Harkin et al., 2009). Traditionally, mission critical electronic systems demanded *design-for-reliability* due to the important function of the system (Ratter, 2004; Vladimirova and Paul, 2009; SEA, 2011). However this design challenge is now penetrating into non-critical systems where engineers must aim to realize reliable systems using unreliable computing fabrics (Barker et al., 2007; Beiu and Ibrahim, 2007; DeSyRe, 2012). Currently, bio-inspired techniques which utilize FPGAs (e.g., Glackin et al., 2004; Negoita and Hintea, 2009) provide adaptive repair but the levels of granularity are still insufficient. Furthermore, such systems also depend upon a central controller to make repair decisions thereby rendering the entire repair process ineffective if it develops a fault.

The present paper draws on numerous published experimental findings and a previous theoretical effort called the astrocyte-neuron (AN) model (Wade et al., 2011a,b) both of which suggest that astrocyte networks provide a more significant role in the function of nervous system than simply structural support. Rather, astrocytes are viewed as regulators of neural circuitry through coordination of transmission at the synapse. Current evidence indicates that retrograde messengers induced in the post-synaptic neuron are fed back either directly or indirectly via astrocyte cells to receptors on the pre-synaptic neuron (Navarrete and Araque, 2010). The present extension of our AN model captures the endocannabinoid interaction between astrocytes and neurons and demonstrates that positive feedback enhances the transmission PR in remote synapses during so-called fault conditions. Our hypothesis is that the emergence of low transmission PR synapses, which result in silent or near silent neurons, is how a “fault” is detected. Essentially a fault is detected when a neuron ceases sustained firing activity. The result is that endocannabinoid release, and therefore both direct and indirect feedback to associated synapses, is stopped or significantly reduced. However, the PR of release at synapses will slowly be enhanced again as a result of other active neuron signaling (e-SP) via astrocytes. Enhancement of the transmission PR of these synapses by indirect retrograde feedback from other active neurons is the proposed repair mechanism.

The self repairing concept minimizes degradation in the information processing capability of the network since the distribution in the weight vector, from the learning phase, is minimally altered by the repair process. If we consider the case where a number of active synapses are severed completely, the repair mechanism redistributes the synaptic weight associated with the lost synapses by increasing PR across the remaining healthy synapses. This is also the case where there is only a partial loss of the synapse. Therefore the repair process simply scales all the weights associated with the faulty neuron. Considering that a single axon can connect to a neuron via many synapses, where information is transmitted across these several synaptic paths with varied strengths/weights, the loss of several synapses will not result in the net information from the pre-synaptic neuron being completely diminished. The repair process will accommodate the loss of synapses by redistributing the original weighting of the lost synapses across the remaining healthy synapses. The result is that information from the pre-synaptic neuron still creates a similar net stimulation of the post-synaptic neuron. While more research is needed on the role of PR in information encoding, we would however, suggest that redistributing PR will result in a “redistribution of information coding” at the level of synapses to reestablish neural depolarization representative of that prior to the faulty condition. More importantly, the repair process exploits existing healthy synapses to take up the signaling effort which was originally sustained by the lost synapses thereby, removing the requirement for redundant synapses. This is a key attribute which enables efficient hardware implementations of the repair mechanism.

Clearly, further research is required to demonstrate self repair at the level of useful large-scale networks as our current network lacks some biological detail. For example, the model used to describe the functional relationship between PR and e-SP/DSE signal requires more experimental evidence to provide a more biophysical model, as does the functional dependency of the 2-AG signal on the activity of the post-synaptic neuron. Furthermore, the sustained firing activity required to create the DSE signal within our model lacks biological realism, firing of this nature is generally only found in motor neurons. However, we purposely implemented our DSE signal in this very simple way due to the lack of a clear understanding of the relationship between DSE and levels of 2-AG. Further investigations are required to underpin biological knowledge about the mechanisms of DSE before our model could faithfully capture the firing patterns found in other brain regions. Moreover, a more complete model would also take account of the spatial distribution of synapses from the delay perspective: delay associated with the astrocyte process. Despite these limitations our self repair network demonstrates the principle for local repair at the level of synapses and therefore provides a building block to develop upon and explore large-scale networks where global repair is possible via an astrocyte network. However, to extend this repairing paradigm a better understanding of astrocyte to astrocyte communication is required. While we are aware that these networks communicate using both gap junctions and ATP (Giaume et al., 2010), no formulation of these communications mechanisms has appeared in the literature. Consequently much research is required to support the modeling of large-scale repair.

### Conflict of interest statement

The authors declare that the research was conducted in the absence of any commercial or financial relationships that could be construed as a potential conflict of interest.

## Appendix

**Table A1:** **Astrocyte parameters**.

**Astrocyte parameter**	**Parameter description**	**Value**
*IP*^*^_3_	Baseline value of IP_3_	0.16 μ M
*r*_IP_3__	Rate of IP_3_ production	0.5 μ M s^−1^
τ_IP_3__	IP_3_ degradation time constant	7 s
τ_Ca_	Decay rate of *f* controlled by level of Cytosolic Ca^2+^	1 s
τ_AG_	Decay rate of 2-AG	10 s
τ_Glu_	Decay rate of Glutamate	100 ms
τ_e_SP__	Decay rate of eSP	40 s
*r*_*C*_	Maximum rate of CICR	6 s^−1^
*r*_*L*_	Ca^2+^ leakage rate from ER	0.11 s^−1^
*r*_Glu_	Maximum rate of Glutamate production	10 μ M s^−1^
*v*_ER_	Maximum rate of SERCA uptake	0.8 μ M s^−1^
*c*_0_	Total free Ca^2+^ cytosol concentration	2 μM
*k*_ER_	SERCA pump activation constant	0.1 μM
*c*_1_	Ratio of ER volume to cytosol volume	0.185
*d*_1_	IP_3_ dissociation constant	0.13 μM
*d*_2_	Ca^2+^ inactivation dissociation constant	1.049 μM
*d*_3_	IP_3_ dissociation constant	0.9434 μM
*d*_5_	Ca^2+^ activation dissociation constant	0.08234 μM
*a*_2_	IP3R Ca^2+^ inactivation binding rate	0.2 μM s^−1^
*m*_eSP_	e-SP weighting factor	55 × 10^3^
Ca^2+^ Threshold	Astrocyte Glutamate release threshold	0.3 μM

**Table A2:** **Astrocyte initial variables**.

**Astrocyte variable**	**Initial value**
Ca^2+^	0.071006 μM
*h*	0.7791
*J*_chan_	0 v
*J*_pump_	0
*J*_leak_	0
*m*_∞_	0
*n*_∞_	0
AG	0
Glu	0
eSP	0
IP_3_	0.16 μM

**Table A3:** **Neuron and synapse parameters**.

**Neuron parameter**	**Parameter description**	**Value**
*v*_th_	Firing threshold voltage	9 mv
*R*_m_	Membrane resistance	1.2 GΩ
τ_mem_	Membrane time constant	60 ms
*I*_inj_	Injected current	6650 pA
